# TNF-α Levels in Respiratory Samples Are Associated with SARS-CoV-2 Infection

**DOI:** 10.1128/spectrum.01411-21

**Published:** 2022-02-09

**Authors:** Matias J. Pereson, Maria Noel Badano, Natalia Aloisi, Roberto Chuit, M. M. E. de Bracco, Patricia Bare

**Affiliations:** a Instituto de Medicina Experimental (IMEX) CONICET, Instituto de Investigaciones Hematologicas (IIHEMA), Academia Nacional de Medicina, Buenos Aires, Argentina; b Department of Microbiology, Immunology, Biotechnology and Genetics, Universidad de Buenos Aires, Facultad de Farmacia y Bioquímica, Instituto de Investigaciones en Bacteriología y Virología Molecular (IBaViM), Buenos Aires, Argentina; c Instituto de Investigaciones Epidemiológicas, Academia Nacional de Medicina, Buenos Aires, Argentina; University of Georgia

**Keywords:** IL-6, SARS-CoV-2, cytokines, tumor necrosis factor

## LETTER

The coronavirus disease 2019 (COVID-19) is one of the current major health concerns. High systemic cytokine levels were correlated with increased morbidity and mortality in COVID-19 patients (1). However, lower plasma cytokine levels were reported in severe COVID-19 compared with other inflammatory conditions (2). Consequently, cytokine contribution in disease progression remains contradictory.

The aim of this study was to evaluate concentrations of IL-6 and TNF-α in swab samples from individuals with symptoms compatible with COVID-19 and analyze their association with SARS-CoV-2 genome presence.

The study was conducted on 127 combined nasopharyngeal and oropharyngeal swabs, referred to the laboratory for detection of SARS-CoV-2. Cytokine levels of symptomatic individuals with detectable (*n* = 52) or undetectable (*n* = 33) SARS-CoV-2 were measured. A group consisting of asymptomatic individuals and negative for SARS-CoV-2 genome (*n* = 42) were included to obtain cytokines’ normal range.

Genomic extraction and real time-based methodology were performed for viral genome detection using the CDC RT-qPCR protocol, 2019-nCov CDC USA. Viral load was calculated considering Ct values.

Concentrations of TNF-α (standard curve range: 0 to 500 pg/mL) and IL-6 (standard curve range: 0 to 300 pg/mL) in swabs were determined using commercial reagents based on enzyme linked immunosorbent assay (BD-Biosciences). Considering the minimum detectable dose of each cytokine and the cross-reactivity with other cytokines in our system, the limit of detection for TNF-α and IL-6 was determined to be 5 pg/mL. All values between 0 and 5, were considered as 5 pg/mL for statistical analysis purposes.

Mann-Whitney U test and chi-square test were used for data analysis. In all cases, a *P value* <0.05 was considered significant.

**FIG 1:**
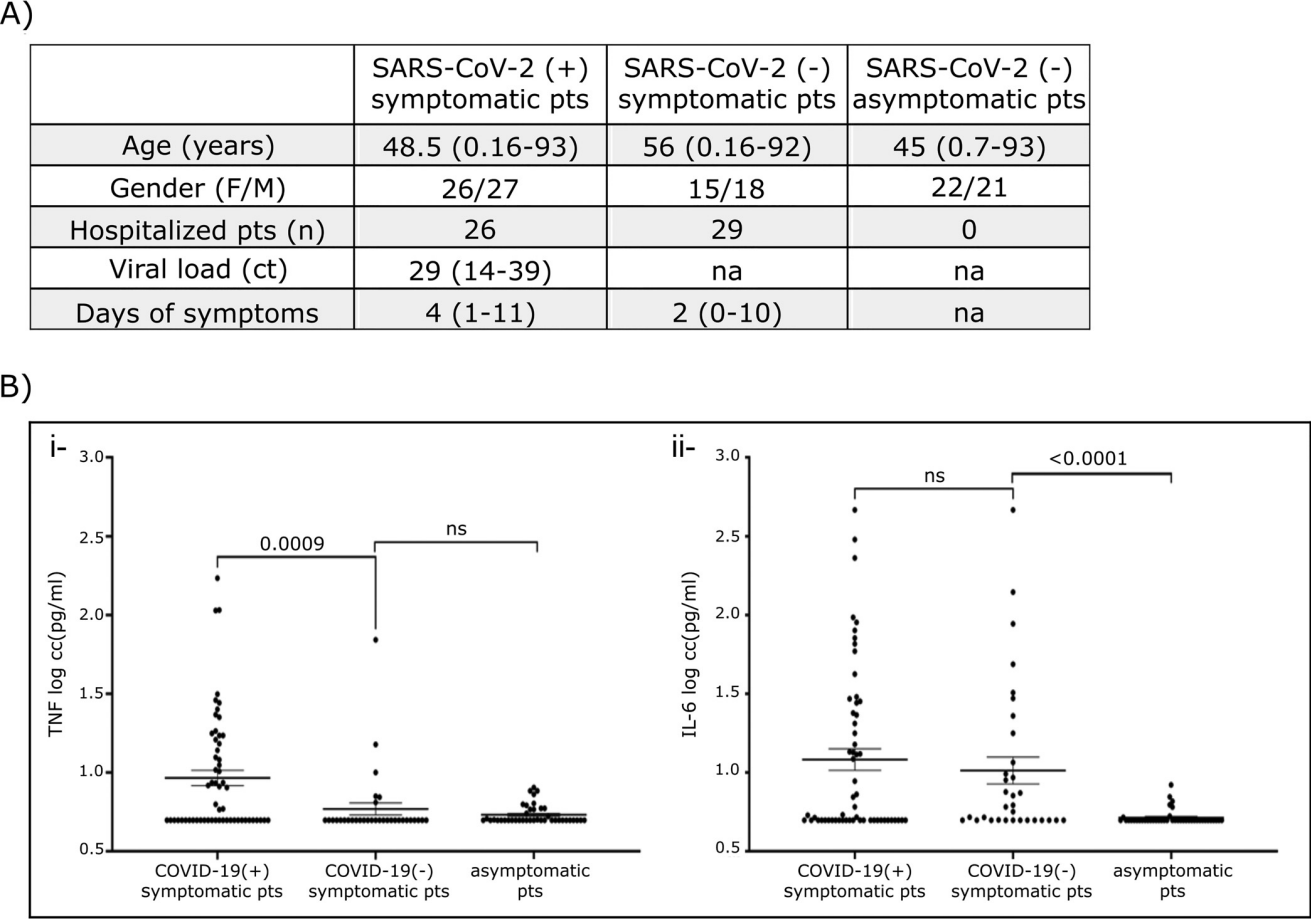
Population characteristics and cytokine concentration in swabs. (A) Population characteristics. Values are expressed as median (range) unless otherwise noted. pts, patients; F, female; M, male; Ct, cycle threshold; na, not applicable. (B) Cytokine levels from SARS-CoV-2 (+) and (–) symptomatic individuals are shown. Mean ± SEM for TNF-α (i) and IL-6 (ii) concentrations are expressed in log (pg/mL). Cytokines were measured in asymptomatic individuals negative for SARS-CoV-2 genome to set up normal ranges. All values below the limit of detection were considered as 5 pg/mL for statistical analysis purposes.

Characteristics of the study population are shown in Fig. 1A. Normal ranges of TNF-α and IL-6 in swab samples are shown in Fig. 1B.

TNF-α levels were higher in SARS-CoV-2 (+) symptomatic group (mean: 16 pg/mL, median value: 5.88 (5 to 172.1) pg/mL) compared with SARS-CoV-2 (–) symptomatic individuals (mean: 7.6 pg/mL, median: 5.00 (5 to 69.9) pg/mL) (*P* = 0.0009) (Fig. 1Bi). IL-6 was increased in some individuals with no significant difference between groups (mean: 33.95 pg/mL, median: 5.40 (5 to 467) pg/mL and for SARS-CoV-2 (–) mean: 30.4 pg/mL, median: 6.07 (5 to 466.6) pg/mL) (Fig. 1Bii). Considering a threshold value of 10 pg/mL for TNF-α, individuals with SARS-CoV-2 (+) were six times more likely to display increased levels of TNF-α (OR = 5.7; *P* = 0.006; 95% CI = 1,551 to 19,11). A correlation between TNF-α or IL-6 levels and viral load (Ct) was not found. Furthermore, age and gender were not related with cytokine concentration among SARS-CoV-2 (+) group. Focusing only on hospitalized patients, individuals with viral genome presence showed increased TNF-α values (mean: 18.7 pg/mL, median 5 pg/mL; 5 to 172.2) compared with those with absent SARS-CoV-2 genome (mean: 7.95 pg/mL, median 5 [5 to 65] pg/mL) (*P* = 0.028).

Cytokine levels have been studied in patients with COVID-19 to analyze their value as biomarkers but also, because cytokine inhibitors have been suggested for the treatment of acute respiratory distress syndrome patients based on the concept of “cytokine storm” in COVID-19 (3). Reports from patients on anti-TNF therapy showed a reduced rate of COVID-19 morbidity and death (4, 5). Our findings, evaluating the concentration of TNF and IL-6 at the entry site of SARS-CoV-2, highlight the pleiotropic role of IL-6 in local inflammation process and show that TNF-α could contribute to disease evolution prognosis as a biomarker. Measurement of TNF-α in nasopharyngeal swabs at diagnosis could identify those individuals that may benefit from immunomodulatory and cytokine inhibiting therapies.
